# Animal Models in Cardiovascular Research: Hypertension and Atherosclerosis

**DOI:** 10.1155/2015/528757

**Published:** 2015-05-03

**Authors:** Xin-Fang Leong, Chun-Yi Ng, Kamsiah Jaarin

**Affiliations:** ^1^Department of Pharmacology, Universiti Kebangsaan Malaysia Medical Centre, Jalan Yaacob Latif, Bandar Tun Razak, Cheras, 56000 Kuala Lumpur, Malaysia; ^2^Department of Clinical Oral Biology, Faculty of Dentistry, Universiti Kebangsaan Malaysia, Jalan Raja Muda Abdul Aziz, 50300 Kuala Lumpur, Malaysia

## Abstract

Hypertension and atherosclerosis are among the most common causes of mortality in both developed and developing countries. Experimental animal models of hypertension and atherosclerosis have become a valuable tool for providing information on etiology, pathophysiology, and complications of the disease and on the efficacy and mechanism of action of various drugs and compounds used in treatment. An animal model has been developed to study hypertension and atherosclerosis for several reasons. Compared to human models, an animal model is easily manageable, as compounding effects of dietary and environmental factors can be controlled. Blood vessels and cardiac tissue samples can be taken for detailed experimental and biomolecular examination. Choice of animal model is often determined by the research aim, as well as financial and technical factors. A thorough understanding of the animal models used and complete analysis must be validated so that the data can be extrapolated to humans. In conclusion, animal models for hypertension and atherosclerosis are invaluable in improving our understanding of cardiovascular disease and developing new pharmacological therapies.

## 1. Introduction

Research animals are valuable tools for understanding the pathophysiology and in developing therapeutic interventions for a disease. These animals are used in basic medical and veterinary research. Various animals have been reported as useful models in studying diseases afflicting humans and animals. Research animals include mice, rats, rabbits, guinea pigs, sheep, goats, cattle, pigs, primates, dogs, cats, birds, fish, and frogs [1]. Concerns have been raised concurrently with the rise of the use of animals over the years. This increase is mainly attributed to the use of genetically altered animals [1]. The similarities and differences between models must be taken into consideration for every project. Careful consideration should be given in choosing the most appropriate animal model to answer the specific research question of the study. With increasing awareness of animal welfare and research ethics, it is important to obtain accurate results using suitable models while reducing wastage of animals used for testing.

Animals are used in biomedical research for the following reasons.

*(i) Feasibility*. Animal models are relatively easy to manage, as compounding effects of dietary intake and environmental factors including temperature and lighting can be controlled. Therefore, there is relatively less environmental variation compared to human studies. Blood vessels and cardiac tissues can be isolated for detailed experimental and biomolecular investigations. Animals typically have a shorter life span than humans. Hence, they make good models, as they can be studied over their whole life cycle or even across several generations [2, 3].

*(ii) Similarities to Human*. Moreover, many animals are suitable due to their similarity in anatomical basis and physiological functions with humans. For example, chimpanzees and mice share about 99% and 98% of DNA with humans, respectively [4, 5]. As a result, animals have the tendency to be affected by many health problems afflicting humans. Therefore, animals are good models for the study of human diseases.

*(iii) Drug Safety*. Preclinical toxicity testing, pharmacodynamics, and pharmacokinetics profile of drugs may be investigated on animals before the compounds or drugs are used in humans. This is vital, as prior to testing on humans, the effectiveness of a drug as potential treatment needs to be carried out on animals [6]. Interventions for diseases must be identified to eventually develop new medicines beneficial to humans and/or other animals. Drug safety profiles need to be determined in order to protect the animals, human, and environment. Harmful and detrimental effects of a drug need to be tested on a whole organism [6]. This can further ensure the dose to be employed in clinical trials, which do not cause fatality in the subsequent studies. The tested chemicals must also be safe for administration and avoid contaminating water, soil, and air. It is unethical to directly test drugs or chemicals on humans, thus warranting the need to use animals in the research, although this has been an issue debated by animal rights and welfare groups.

Before conducting research on animals, researchers must ensure that animals are essential for their experiments, with no viable alternatives. The use of 3Rs principle relating to animal research has been a practice since first introduced by Russell and Burch in 1959 [7]. The 3Rs refer to replacement, reduction, and refinement. Replacement means conducting experiments using nonanimal models, such as* in vitro* method with cell culture as well as with computer model simulation (*in silico*), whenever possible. Nevertheless, the information obtained from* in vitro* is typically limited when compared to* in vivo* studies. Reduction refers to the need to reduce the number of animals, either from previous studies or by using calculation of size sample with a good experimental design. Refinement refers to efforts to minimize pain and suffering of test animals, taking into consideration animal handling and surgical procedures, housing environment and living conditions, and improvements in animal husbandry. These 3Rs are aimed at providing humane and scientifically improved research involving or avoiding the use of animal models [8]. Guidelines for reporting animal study are available to ensure the justification of using animals, such as the Animals in Research: Reporting In Vivo Experiments (ARRIVE) guidelines [9] and the Gold Standard Publication Checklist (GSPC) [10].

Even though animal studies have contributed much to our understanding of mechanisms of diseases, their value in predicting the effectiveness of treatment strategies in clinical trials has remained controversial [11–13]. Clinical trials are essential, as animal studies do not predict with sufficient certainty what will happen in humans. Hence, the findings from animal studies may not be deemed suitable for extrapolation to humans. A report by Williams et al. [11] suggested that a recurrent failure of interventions to translate the results obtained in animal studies to the clinical settings may be due to the ability to control genetic background in animal studies. Controlling the genetic background produces more consistent results. Additionally, it is possible that some of the genetic effects of the candidate loci are context-dependent. For example, the specific loci may play a significant role in sex (male versus female) or in age (young versus old) or in people of a specific body mass index or race [12, 13]. Since these characteristics are not usually investigated or analyzed in many of the studies, there is a possibility that the failure to replicate is due to interactions between genes and environmental factors as well as to gene-gene interactions. If there are interactions between environmental risk factors and genotypes, the validity of extrapolation may become complicated [14].

Moreover, this failure may be explained in part by the methodological flaws in animal studies, eventually leading to a systematic bias which might generate incorrect conclusions about efficacy of a drug or a compound [11]. Per Bracken [15], reasons for the failure of animal experiments which may be translated into human trials include poor experimental design, execution, and analysis [16]. Systematic reviews provide information on whether animal studies are being properly carried out and published. However, systematic reviews are not able to resolve all queries regarding the applicability and relevance of animal studies to humans [13]. Selection biases affect how literature is selected and subsequently included in the systematic review, due to the criteria set by the different authors. The objectives of animal experiments are typically to discover new knowledge or make advances in understanding the diseases, instead of predicting the outcomes of human trials. Therefore, data obtained from animal studies may be unsuitable or too diverse for meaningful comparison with and prediction of the results of human trials. Nevertheless, systemic reviews ensure that all animal studies are published regardless of outcome, in order to avoid unnecessary duplication of expensive animal experiments [17]. Furthermore, systematic reviews may improve the quality and translational value of animal research to human trial [18].

## 2. Animal Models for Hypertension

Hypertension is one of the major risk factors for cardiovascular diseases. It has become a major public health issue in most developed and developing countries [19–21]. According to the* Seventh Report of the Joint National Committee on Prevention, Detection, Evaluation and Treatment of High Blood Pressure*, high blood pressure (BP) is defined as systolic blood pressure (SBP) greater than 140 mmHg and/or diastolic blood pressure (DBP) greater than 90 mmHg [22]. Patients with SBP ranging between 120 mmHg and 139 mmHg, or DBP of 80 mmHg to 89 mmHg, are categorized as prehypertensive. They have a higher risk of developing hypertension and therefore require medical intervention [22].

Human essential hypertension is a complex multifactorial disease which is influenced by genetic and environmental factors. Various models of experimental hypertension have been primarily developed to mimic hypertensive responses observed in humans [23]. These models are beneficial in the pharmacological screening of potential antihypertensive drugs, in addition allowing researchers to have a better understanding of the etiology, development, and progression of hypertension [24]. Since animal models of hypertension are a mimicry of human hypertension, many of these models have been developed using the etiological factors which have been hypothesized to have a contributory role in human hypertension, such as excessive salt intake, hyperactivity of renin-angiotensin-aldosterone system (RAAS), and genetic predisposition [24]. One animal model is insufficient for explaining the antihypertensive effects of a particular drug, because many pathways are involved in the development of BP dysregulation. In another word, several animal models are required to examine particular cardiovascular changes in an effective study [25]. Therefore, it is advisable that each of the studied models explains a unique pathway in the development of hypertension.

Several criteria need to be considered in order to develop an ideal animal model for hypertension. These factors include the feasibility and size of the animals, the reproducibility of the model, the ability to predict the potential antihypertensive properties of a drug, the similarity to human disease (mode of the disease: slow on-set versus acute), and economical, technical, and animal welfare considerations [23, 24]. In the past, dogs were mostly employed as a model to study hypertension. Currently, the preferred animal model is the rat. Along with rats, occasionally mice, monkeys, and pigs are also used as a model for experimental hypertension [26, 27]. These species have not been studied extensively for both practical and financial reasons. In 1963, Okamoto and Aoki introduced an experimental hypertension model without the involvement of physiological, pharmacological, or surgical intervention [28]. This model is known as the spontaneously hypertensive rat (SHR), which is the genetic strain of hypertensive rat. SHR has become the animal of choice for the screening of antihypertensive agents and the cornerstone of medical research in experimental hypertension [29].

Several forms of murine genetic models, including SHR, have become the focus of hypertensive research. The short life span, small size, and relatively low cost of the animals enable the researchers to study the natural history, genetic factors, and pathophysiological changes in hypertension [29]. Other strains have been developed, including the New Zealand strain [30], Milan strain [31], Dahl salt-sensitive strain [32], Sabra strain [33], and Lyon strain [34]. Essential hypertension is the most frequently encountered human type of hypertension. It is also known as primary hypertension, contributing to 95% of incidences. Essential hypertension is associated with genetic influences. Among the many strains of rat models SHR is generally used, even though it represents only a particular type of hypertension [35].

In addition to the genetic type of animal models, renovascular hypertension is a commonly employed model of hypertension. RAAS plays pivotal role in this form of hypertension [36, 37]. In 1934, Goldblatt et al. developed a hypertension model through partial constriction of the renal artery in dog [38]. This has led to other renal-induced hypertension model using rats, rabbits, sheep, and cats [39]. When the renal artery is ligated or constricted, RAAS and the sympathetic nervous system are activated [40]. Renin is secreted by the kidneys when sympathetic activity is enhanced. Angiotensinogen is converted to angiotensin-I (Ang I) in the presence of renin. Angiotensin-converting enzyme (ACE) plays a vital role in the regulation of BP via hydrolysis of the inactive form of Ang I to the active form, angiotensin II (Ang II). ACE is mainly located on the surface of the endothelium and epithelium involved in the constriction of blood vessels, subsequently leading to elevation of BP. Ang II is a potent vasoconstrictor and affects cardiovascular homeostasis. Apart from the role in vasoconstriction, Ang II also stimulates the release of aldosterone, further increasing blood volume and BP due to water and salt retention [41].

Nitric oxide (NO) has been demonstrated to be a potent vasodilator, and its release from the endothelium may be triggered by vasoactive substances such as acetylcholine (ACh) [42]. The endothelium preserves its integrity through endothelium-relaxing dependent factor, which is the best to be characterized as NO [43]. Therefore, NO plays an important role in the regulation of BP [44]. The production of NO is catalyzed by nitric oxide synthase (NOS). Deficiency of NOS has led to a reduction in NO synthesis [45, 46]. Impaired NO bioavailability will result in reduced endothelium-dependent vasorelaxation, eventually leading to hypertension. This NO-deficient model can be induced by oral administration of N^*ω*^-nitro-L-arginine methyl ester (L-NAME) up to eight weeks, resulting in a significant rise in both SBP and DBP, renal and hepatic markers, and inflammatory parameters in male Wistar rats [47]. Often, BP is elevated after four weeks of L-NAME treatment. Long-term administration of NOS inhibitors, such as L-NAME, provides a new form of hypertension with target organ damage. Studies have reported that L-NAME-induced hypertension has been associated with attenuated endothelium-dependent relaxations, cardiac and aortic tissue damage, renal vascular, and glomerular fibrosis [48–50]. Since the etiology of hypertension is different among the various animal models, it is imperative to make a rational choice for a specific model (Table 1). The choice will significantly affect the outcome of the study.

Soriguer et al. [51] conducted a study on cooking oils, reporting that repeatedly oxidized frying oil is an independent risk factor for hypertension. Hence, hypertension is related to the degradation of the dietary frying oil. Previously, adult male Sprague-Dawley rats aged 3 months were administered with 15% weight/weight (w/w) of repeatedly heated vegetable oils for 16 weeks [52] or 24 weeks [53–56]. Chronic consumption of heated oil diets causes an increase in BP. The BP-raising effect of the heated vegetable oils may be attributable to the diminished endothelium-dependent relaxation responses. Heated oil diet promotes oxidative stress, resulting in NO sequestration and inactivation. Furthermore, heated oil causes a significant increase in ACE activity and a reduction in heme oxygenase content. The thermal oxidation of vegetable oils promotes the generation of free radicals and may contribute to the pathogenesis of hypertension in rats. This heated oil-induced hypertension model employed male instead of female rats. Female hormones have been shown to have cardioprotective properties [57, 58]. BP was measured using the conventional heating tail-cuff method. Even though invasive methods such as carotid arterial cannulation may provide more accurate readings, these may cause injury in the animals and further complicated the experiment. In addition, these studies were performed to compare and monitor the effects of heated oil diets among the experimental groups up to 24 weeks using large number of rats. Thus, the noninvasive tail-cuff method is more suitable for measuring BP for long-term studies [59].

## 3. Animal Models for Atherosclerosis

Atherosclerosis, or “hardening of the arteries,” is a chronic inflammatory disease characterized by endothelial dysfunction and disorganization of intimal architecture owing to the accumulation of lipid deposits, inflammatory cells and cell debris in the intima of elastic, and medium to large muscular arteries. It underlies many of the common causes of cardiovascular deaths, including stroke and heart attack [85]. Several modifiable (including advanced age, gender, and heredity) and nonmodifiable risk factors (including dyslipidemia, hypertension, sedentary lifestyle, tobacco smoking, and diabetes mellitus) have been identified for the development of atherosclerosis [86]. Many clinical and experimental attempts have been performed to understand the pathophysiology of the disease. Amongst them, animals have been used for more than a century to study atherosclerosis. The first evidence that experimental atherosclerosis could be induced in animals came into view as early as 1908 by Ignatowski, who demonstrated atherogenesis in the aortic wall of rabbits fed a diet enriched in animal proteins including meat, eggs, and milk [87]. Since then, numerous animal models have been used for understanding the mechanisms involved in both induction and regression of atherosclerotic lesions [88, 89]. Rats, rabbits, dogs, pigs, and monkeys are well-established animal models for atherosclerosis, and thrombosis. Nonhuman primates, hamster, mouse, cat, and guinea pig have also been used, but with lesser extent [90].

Several studies documented a significant relationship between elevated levels of serum cholesterol and development of atherosclerotic plaques in experimental animals. High-fat diets such as the 1% or 2% cholesterol diet have been found to elevate serum low-density lipoprotein (LDL), inducing atherogenesis in certain animals such as hamsters [91] and guinea pigs [92]. Therefore, the use of high-fat diets in promoting atherosclerosis in animal models has been a valuable tool for studying pathogenesis, as well as for testing potential therapies in reversing the atherosclerotic process.

Overall, an ideal animal model should be representative of the human atherosclerosis and should be feasible and affordable. Although animal models have played a significant role in our understanding of induction of atherosclerotic lesions, they have some limitations (Table 2). Not all experimental animals, such as rats and mice, respond similarly to a given high-fat diet, due to inherent genetic differences. Rats and mice are not good models for atherosclerosis, because they are typically resistant to atherogenesis, and even diets as high as 10% w/w cholesterol are not usually sufficient to produce vascular lesions [121]. The lipid metabolism of a normal rat and a mouse is primarily based on high-density lipoprotein (HDL) rather than on LDL as in humans, which might be attributable to resistance to atherogenesis [122]. The use of other interventions, such as vitamin D_3_, to establish atherosclerotic calcification or aortic medical calcification, is often required [123]. Furthermore, a high-fat diet may represent a toxic proinflammatory stimulus rather than a low and chronic inflammatory state to animals [124]. Moreover, from a nutritional perspective, the dilution of a chow diet with lipids may increase the caloric density of the diet and reduce the ratio of essential nutrients to dietary energy, potentially leading to an imbalance in nutrient intake in animals consuming the atherogenic diet [125].

A small, genetically reproducible, murine model of atherosclerosis has been long desired due to projections of relatively easy handling and breeding procedures as well as its low cost. Researchers have used genetic technology to produce a number of genetically modified murine models to overcome the many deficiencies of larger animals, particularly to allow studies of potential therapies that require large numbers of subjects. An exciting scientific breakthrough occurred in 1992, when Zhang et al. found that ApoE-deficient mice generated by gene targeting had five times higher plasma cholesterol level and developed foam cell-rich depositions in their proximal aortas by the age of 3 months [114]. This model was the very first line of genetically modified murine model for atherosclerosis studies introduced to the research community. Since then, further research has led to other genetically modified models that mimic important aspects of atherosclerosis, such as fatty streaks, deposition of foam cells, vulnerable and stable plaques, and related complications such as arterial calcification, ulceration, hemorrhage, plaque rapture, thrombosis, and stenosis. Fatty Zucker rats, cholesterol ester transfer protein (CETP) transgenic rats, LDL receptor-knockout (KO) mice, and* db/db* mice are a few of the genetically modified models developed over recent years (Table 3). The development of techniques for direct genetic modification that have been previously restricted to murine species is promising to produce other new strains.

According to the oxidation hypothesis of atherosclerosis [126], oxidized LDL (oxLDL) plays a key role in the initiation of the atherosclerotic lesion as well as in almost every step of the atherogenic process, from the formation of cholesterol-laden foam cells in plaques to the functioning as chemoattractants for macrophages and vascular smooth muscle cells [126, 127]. Since the etiology of atherosclerosis is multifactorial, the potential lipid-raising effect and lipid oxidation might contribute to atherogenesis together. The potential atherogenic effect of heated oils has been studied in experimental animals. Staprãns et al. [128] reported an increase of *β*-very low-density lipoprotein (*β*-VLDL) fraction and the formation of fatty streak lesions in aortas in male New Zealand White rabbits fed a low-cholesterol (0.25%) diet containing 5% thermal-oxidized corn oil. Atherosclerotic lesions have also been observed in genetically modified murine models, that is, LDLR^−/−^ and apoE^−/−^ mice after chronic consumption of an oxidized cholesterol diet [129].

However, there are still limitations in the experimental animals used in the aforementioned studies. For instance, cholesterol diets in rabbits may lead to hepatic toxicity [96]. Furthermore, genetically modified mice are rather costly and may impose a substantial financial constraint to a research as well as limit the number of samples. Therefore, a more feasible and affordable alternative has been developed to induce atherosclerosis in rats. Adult female Sprague-Dawley rats were ovariectomized prior to 16-week administration of 2% cholesterol diet fortified with 15% w/w of heated vegetable oil [130–132]. Ovariectomy was performed to simulate a postmenopausal condition characterized by the absence of cardioprotective estrogen [133].

Although there was a trend of increasing total cholesterol (TC) in all oil-fed groups, only heated oil-treated rats showed significant increase in serum TC compared to the control [130, 131]. There were pronounced focal disruptions in the aortic intimal layer of the rats fed heated oil. Moreover, mononuclear cells were also observed in the intimal layer [132]. Based on the findings, it is possible to overwhelm rats' natural resistance to atherosclerosis by removing the ovaries. A further attempt was made to induce atherosclerosis in male Sprague-Dawley rats, as the use of the previous ovariectomized models is confined to menopause-induced atherosclerosis. Rats fed with standard rat chow fortified with 15% w/w of heated oil for 16 to 24 weeks. Histological study of the heart revealed cardiac toxicity with the presence of necrosis in cardiac tissue [52]. The intimal layer was observed to be noticeably thickened due to a massive lipid accumulation in the subendothelial space [134]. Lectin-like oxidized low-density lipoprotein receptor-1 (LOX-1), an endothelial receptor for endocytosis of oxLDL, was significantly increased in heated oil-fed rats compared to the control [135]. There were significant positive correlations between LOX-1 and the expressions of vascular cell adhesion molecule-1 (VCAM-1) and intercellular adhesion molecule-1 (ICAM-1) in heated oil-fed rats [135]. We suggest that heated oil diet can be used to induce atherosclerosis in rat models. However, the atherosclerosis-inducing effect seems to be more prominent in the ovariectomized rats than in male rats, as a longer period of intervention is required in male animals. Though the male rat model requires a longer duration of diet treatment to develop atherosclerosis, it is free from any surgical intervention in contrast to female rats undergoing ovariectomy. The use of other interventions such as vitamin D_3_ [123] may be helpful in the escalation of atherosclerotic plaque formation.

## 4. Conclusion

Progress in cardiovascular disease control requires understanding of the pathogenesis of the disease and testing of potential therapies, both experimentally and clinically. Experimental animal models, particularly murine species, have been a useful tool in this regard. The ideal animal model of cardiovascular disease should be representative to human conditions metabolically and pathophysiologically. The development of genetically modified animal models has enabled researchers to manipulate a specific target (either gene or protein), the role of which in pathogenesis may be subsequently established. This has led to the discovery of a vast spectrum of potential targets for ameliorative intervention. While the use of animal models has undeniably offered novel insights into different important aspects of a disease, still there are no species which are absolutely suitable for all studies, given the multifactorial nature of cardiovascular disease. Therefore, it is of utmost importance to choose an appropriate model to study different parts of cardiovascular disease. Otherwise, many exciting research findings may fail when translating into human studies. An agreement on appropriate experimental models for the study of different facades of cardiovascular disease would be a viable and effective strategy to further the advancement in this field.

## Figures and Tables

**Table 1:** Common animal models for hypertension with different etiology.

Experimental model	Description
Genetic hypertension (i) SHR (ii) Dahl salt-sensitive (iii) Transgenic	(i) SHR is developed by inbreeding Wistar rats (brother-to-sister) with the highest BP [28]. The BP increases at week 4 to week 6 and reach systolic BP of 180–200 mmHg [28]. SHR may develop cardiac hypertrophy, cardiac failure, renal dysfunction, and impaired endothelium-dependent relaxations [60–62]. (ii) Dahl salt-sensitive rats derived from Sprague-Dawley rats on the basis of administering high NaCl diet. Salt-sensitive rats become hypertensive when given normal salt diets; however these rats develop severe and fatal hypertension with high salt diet (8% NaCl) [32]. These rats may develop cardiac hypertrophy, severe cardiac failure, hypertensive nephropathy, impaired endothelium-dependent relaxations [63–65]. (iii) Transgenic model can be generated by overexpression of a specific gene, for example, the mouse Ren-2 gene, and TGR(mREN2)27 [66]. Manifestations include marked cardiac hypertrophy, moderate proteinuria, and impaired endothelium-dependent relaxations [67, 68].

Endocrine hypertension	(i) Administration of DOCA in a combination with high salt diet and unilateral nephrectomy [69]. (ii) DOCA-induced hypertension induces a low renin model of hypertension [70]. (iii) Increased cardiac weight, proteinuria, glomerulosclerosis, and impaired endothelium-dependent relaxations [71, 72].

Environmental hypertension	(i) Stress-induced hypertension using flashing lights, loud noise, restraint cage, and cold or hot stimuli [73, 74]. (ii) Activation of sympathetic nervous system and RAAS may contribute to the initiation of stress-induced hypertension [75, 76].

Pharmacological hypertension	(i) Nitric oxide-deficient model by administering NOS inhibitors such as L-NAME [77]. (ii) Increase in BP was reported during long-term oral treatment with NOS inhibitors [78, 79]. (iii) Development of endothelial dysfunction is gradually with increased of BP [80].

Renal hypertension	(i) This includes two-kidney one-clip hypertension (2K1C; constriction of one renal artery while the contralateral kidney is left intact), one-kidney one-clip hypertension (1K1C; one renal artery is constricted and the contralateral kidney is removed), and two-kidney two-clip hypertension (2K2C; constriction of aorta or both renal arteries) [81, 82]. (ii) In the two-kidney model, circulating renin and aldosterone levels are increased [83], which are most notably in the early phase of hypertension [84].

SHR: spontaneously hypertensive rat; BP: blood pressure; NaCl: sodium chloride; TGR: transgenic rat; RAAS: renin-angiotensin-aldosterone system; DOCA: deoxycorticosterone acetate; NOS: nitric oxide synthase; L-NAME: N^*ω*^-nitro-L-arginine methyl ester.

**Table 2:** Advantages and disadvantages of common animal models for atherosclerosis.

Animal	Advantages	Disadvantages
Rats and mice	(i) Low cost (ii) High availability (iii) Easy to handle and maintain (iv) Manageable breeding (v) Well-established genomic sequencing permit genetic manipulation	(i) Typically resistant to atherogenesis (ii) Absence of plasma CETP activity [93] (iii) Most cholesterol is transported through HDL particles [94] (iv) The small size of mice limits frequent blood sampling and dissection of small arteries

Rabbits	(i) Easy to handle and maintain (ii) Relatively inexpensive (iii) High availability (iv) Sensitive to dietary cholesterol induction of atherosclerosis (v) Large enough to permit physiological experiments	(i) Lesion location less compared with humans [95] (ii) Deficiency in hepatic lipase leads to hepatotoxicity following prolonged cholesterol feeding [96]

Pigs	(i) An anatomically and physiologically similar cardiovascular system compared to humans [97] (ii) Susceptible to spontaneous atherosclerosis [98] (iii) Comparable patterns of plaque distribution [99] (iv) High availability (for miniature pigs)	(i) Large size with resultant management difficulties (ii) High maintenance cost

Dogs	(i) Easy to work with (ii) Ideal size (iii) High availability	(i) Highly resistant to atherogenesis (ii) Status and anthropomorphic attitudes toward dogs (iii) Differences in important aspects of their cardiovascular system than humans [100]

Hamsters	(i) Low cost (ii) High availability (iii) Easy to handle and maintain (iv) Carry a significant portion of its plasma cholesterol in LDL particles and is therefore close to humans [101] (v) Sensitive to high-fat diets [102]	(i) Inconsistency of lesion development and absence of advanced lesions [103] (ii) Require highly abnormal diets and/or treatment with a cytotoxic chemical agent, such as streptozotocin [104]

Guinea pigs	(i) Develop diet-induced atherosclerosis (ii) Most of cholesterol is transported in LDL particles [105] (iii) Ovariectomized guinea pigs showed a similar plasma lipid profile as in postmenopausal women [106]	(i) Require constant supplementation with vitamin C, which potentially acts as an antioxidant to interfere with atherogenesis [107]

Nonhuman primates	(i) Genetically resemblance to humans (ii) Similar omnivorous diet (iii) Similar metabolism (iv) Develop metabolic syndrome as they age [108]	(i) Expensive (ii) Low availability (iii) Live long (thus requiring lengthy experimental periods) (iv) Potential carriers of dangerous viral zoonoses [104] (v) Significant ethical issues

Pigeon	(i) Low cost (ii) Easy handling (iii) Susceptible to atherosclerosis (iv) Sufficient size	(i) Nonmammalian (ii) Lipoprotein compositions and metabolism are different [109] (iii) Differences in arterial histology [110]

Chicken	(i) Low cost (ii) High availability (iii) Develop atherosclerosis naturally in aorta and coronary arteries, with cholesterol feeding accelerating the pathogenesis [111]	(i) Nonmammalian (ii) Viral infection is associated with atherosclerosis [112, 113]

CETP: cholesterol ester transfer protein; HDL: high-density lipoprotein; LDL: low-density lipoprotein.

**Table 3:** Genetically modified animal models for atherosclerosis.

Experimental model	Description
Apolipoprotein E knockout (ApoE^−/−^) mice	Apolipoprotein E (apoE), a constituent of lipoprotein responsible for packaging cholesterol and other fats and carrying them through the bloodstream, is inactivated by gene targeting. They exhibit a higher total plasma cholesterol concentration of 11 mM compared to 2 mM in their parent C57BL/6 mice [114].

LDL receptor knockout (LDLR^−/−^) mice	LDL receptor (LDLR) is a cell surface receptor in liver cells that mediates the endocytosis of apoE to clear cholesterol-abundant LDL particles from the circulation. Total plasma cholesterol levels increase twofold compared to those of wild-type, owing to a seven- to ninefold increase in intermediate density lipoproteins (IDL) and LDL without a significant change in HDL [115].

Scavenger receptor class B member 1 knockout (SR-BI KO) mice	Scavenger receptor class B member 1 (SR-BI) functions in facilitating the uptake of cholesterol from HDL in the liver. It plays a key role in determining the levels of plasma cholesterol (primarily HDL). Heterozygous and homozygous mutants show 31% and 125% increase, respectively, in plasma cholesterol concentrations than wild-types [116].

*db/db* mice	OB-R is a high affinity receptor for leptin, an important circulating signal for the regulation of feeding, appetite, and body weight. Fatty acid oxidation rates are progressively higher in *db/db* mice in parallel with the earlier onset and greater duration of hyperglycemia [117].

*ob/ob* mice	A mutation results in a structurally defective leptin that does not bind to the OB-R. Mice that are *ob/ob* have no leptin action and exhibit obesity and endothelial dysfunction [118].

Fatty Zucker rats	A spontaneous mutant gene (*fa* or fatty) that affects the action of the leptin. They have high levels of lipids and cholesterol in their bloodstream and become noticeably obese by 3 to 5 weeks of age and over 40% lipid of their body composition by 14 weeks of age [119].

Cholesterol ester transfer protein (CETP) transgenic rats	CETP inhibits HDL-mediated reverse cholesterol transport by transferring cholesterol from HDL to very low-density lipoprotein (VLDL) and LDL, promoting atherogenesis. The animals exhibit 82% increase in non-HDL cholesterol in addition to 80% reduction in HDL cholesterol when compared to wild-type rats [120].

HDL: high-density lipoprotein; LDL: low-density lipoprotein.
